# Palmitate-Induced Cardiac Lipotoxicity Is Relieved by the Redox-Active Motif of SELENOT through Improving Mitochondrial Function and Regulating Metabolic State

**DOI:** 10.3390/cells12071042

**Published:** 2023-03-29

**Authors:** Carmine Rocca, Anna De Bartolo, Rita Guzzi, Maria Caterina Crocco, Vittoria Rago, Naomi Romeo, Ida Perrotta, Ernestina Marianna De Francesco, Maria Grazia Muoio, Maria Concetta Granieri, Teresa Pasqua, Rosa Mazza, Loubna Boukhzar, Benjamin Lefranc, Jérôme Leprince, Maria Eugenia Gallo Cantafio, Teresa Soda, Nicola Amodio, Youssef Anouar, Tommaso Angelone

**Affiliations:** 1 Cellular and Molecular Cardiovascular Pathophysiology Laboratory, Department of Biology, Ecology and Earth Sciences (DiBEST), University of Calabria, 87036 Rende, Italy; 2 UNIROUEN, Inserm U1239, Neuroendocrine, Endocrine and Germinal Differentiation and Communication (NorDiC), Rouen Normandie University, 76000 Mont-Saint-Aignan, France; 3 Department of Physics, Molecular Biophysics Laboratory, University of Calabria, 87036 Rende, Italy; 4 CNR-NANOTEC, Department of Physics, University of Calabria, 87036 Rende, Italy; 5 STAR Research Infrastructure, University of Calabria, Via Tito Flavio, 87036 Rende, Italy; 6 Department of Pharmacy, Health and Nutritional Sciences, University of Calabria, 87036 Rende, Italy; 7 Centre for Microscopy and Microanalysis (CM2), Department of Biology, Biology, Ecology and Earth Sciences (DiBEST), University of Calabria, 87036 Rende, Italy; 8 Endocrinology, Department of Clinical and Experimental Medicine, University of Catania, Garibaldi-Nesima Hospital, 95124 Catania, Italy; 9 Department of Health Science, University Magna Graecia of Catanzaro, 88100 Catanzaro, Italy; 10 UNIROUEN, UMS-UAR HERACLES, PRIMACEN, Cell Imaging Platform of Normandy, Institute for Research and Innovation in Biomedicine (IRIB), 76183 Rouen, France; 11 Department of Experimental and Clinical Medicine, Magna Graecia University, 88100 Catanzaro, Italy; 12 National Institute of Cardiovascular Research (INRC), 40126 Bologna, Italy

**Keywords:** antioxidants, cardiomyocyte, lipotoxicity, peptides, selenoproteins

## Abstract

Cardiac lipotoxicity is an important contributor to cardiovascular complications during obesity. Given the fundamental role of the endoplasmic reticulum (ER)-resident Selenoprotein T (SELENOT) for cardiomyocyte differentiation and protection and for the regulation of glucose metabolism, we took advantage of a small peptide (PSELT), derived from the SELENOT redox-active motif, to uncover the mechanisms through which PSELT could protect cardiomyocytes against lipotoxicity. To this aim, we modeled cardiac lipotoxicity by exposing H9c2 cardiomyocytes to palmitate (PA). The results showed that PSELT counteracted PA-induced cell death, lactate dehydrogenase release, and the accumulation of intracellular lipid droplets, while an inert form of the peptide (I-PSELT) lacking selenocysteine was not active against PA-induced cardiomyocyte death. Mechanistically, PSELT counteracted PA-induced cytosolic and mitochondrial oxidative stress and rescued SELENOT expression that was downregulated by PA through FAT/CD36 (cluster of differentiation 36/fatty acid translocase), the main transporter of fatty acids in the heart. Immunofluorescence analysis indicated that PSELT also relieved the PA-dependent increase in CD36 expression, while in SELENOT-deficient cardiomyocytes, PA exacerbated cell death, which was not mitigated by exogenous PSELT. On the other hand, PSELT improved mitochondrial respiration during PA treatment and regulated mitochondrial biogenesis and dynamics, preventing the PA-provoked decrease in PGC1-α and increase in DRP-1 and OPA-1. These findings were corroborated by transmission electron microscopy (TEM), revealing that PSELT improved the cardiomyocyte and mitochondrial ultrastructures and restored the ER network. Spectroscopic characterization indicated that PSELT significantly attenuated infrared spectral-related macromolecular changes (i.e., content of lipids, proteins, nucleic acids, and carbohydrates) and also prevented the decrease in membrane fluidity induced by PA. Our findings further delineate the biological significance of SELENOT in cardiomyocytes and indicate the potential of its mimetic PSELT as a protective agent for counteracting cardiac lipotoxicity.

## 1. Introduction

Obesity represents a major public health problem that seriously increases the risk of developing cardiovascular diseases (CVD) and directly contributes to incident cardiovascular risk factors [1]. In obese patients, the increased left ventricular mass and myocardial changes correlate with adiposity, which is responsible for cardiac steatosis by promoting the ectopic deposition of triglycerides in the heart [2]. Following lipid accumulation within the cells of the cardiovascular system, several complex mechanisms drive myocardial dysfunction, leading to heart failure (HF). In this process, known as cardiac lipotoxicity, saturated long chain fatty acids (FAs)–such as palmitic acid (PA)–play a major role due to their ability to alter cellular structures, triggering oxidative stress, endoplasmic reticulum (ER) stress, defective insulin signaling, mitochondrial dysfunction, inflammation, and myofibrillar dysfunction, culminating in cell death [3,4]. Despite the high metabolic flexibility of the heart in terms of substrate utilization, as well as its capacity to adjust the rate of FA uptake to match myocardial demand to obtain energy [5], the augmented circulating levels of free FAs during obesity dramatically increase the myocardial uptake of lipids that may be stored as triglycerides. However, since cardiomyocytes possess a limited storage capacity, the excess free FAs are also shunted into non-oxidative pathways, leading to myocardial lipotoxic injury [6]. Accordingly, elevated serum levels of PA, which represents the major circulating saturated FA, have been proposed as a potential factor contributing to lipotoxic cardiomyopathy development [7]. During chronic lipid overload conditions, excessive cytosolic FAs can increase mitochondrial uncoupling, which in turn may generate reactive oxygen species (ROS) and activate stress-sensitive pathways, leading to oxidative stress [6]. Additionally, ER protein misfolding secondary to the excess of lipids also promotes ER and nuclear oxidative stress [8]. Therefore, endogenous antioxidant enzymes are crucial to detoxify lipid hydroperoxides and other reactive species to maintain the normal cellular machinery, in particular during lipid overload conditions. In this context, selenoproteins are considered among the most potent antioxidant defense systems, with crucial implication in several pathophysiological contexts, including CVD [9,10]. Selenoprotein T (SELENOT) is an ER-resident thioredoxin-like enzyme and the only ER-located selenoprotein whose gene knockout in mice is lethal early during embryogenesis [11]. As a member of the selenoprotein family, SELENOT contains a selenocysteine (Sec, U) residue in its CVSU amino acid redox motif that is fundamental for its biological functions, including the regulation of ER homeostasis, *N*-glycosylation, and intracellular Ca^2+^ mobilization and neuroendocrine secretion. We previously showed in transgenic cell and animal models that a reduced expression of SELENOT is associated with oxidative and nitrosative stress, unfolded protein response (UPR) activation, and the depletion of Ca^2+^ stores leading to altered hormone secretion [12,13]. Considerable interest has also arisen in the recent years regarding the biological significance of SELENOT in glucose homeostasis and cardiac pathophysiology. SELENOT is strongly expressed in human and mouse pancreatic β- and δ-cells, and conditional pancreatic β-cell SELENOT-knockout mice exhibited impaired glucose tolerance with a deficit in insulin production/secretion [14]. On the other hand, SELENOT is highly expressed during the early hyperplastic growth of cardiomyocytes, suggesting its involvement in cardiac development during embryogenesis, and can also protect cardiomyocytes following myocardial ischemia/reperfusion (MI/R) insult [15,16].

The biological impact of SELENOT in cardiac and metabolic pathophysiology raised an intriguing hypothesis concerning its role in protecting cardiomyocytes exposed to a dysmetabolic condition generated by lipid overload. More precisely, considering that the CVSU motif of SELENOT carries a selenosulfide oxidoreductase that interacts with other cellular components via redox reactions and is thus essential for SELENOT biological action, in the present study, we designed a small SELENOT mimetic peptide (called PSELT), encompassing the redox motif of the full protein, and tested its ability to protect H9c2 cardiomyocytes against PA-induced lipotoxicity.

## 2. Materials and Methods

### 2.1. Peptides and Drugs

The SELENOT-derived peptide 43–52 (corresponding to the sequence H-Phe-Gln-Ile-Cys-Val-Ser-Sec-Gly-Tyr-Arg-OH) designated as PSELT and its inactive form (Ser 46,49, designated as the inert-PSELT or I-PSELT) were chemically synthesized as described previously [17]. Dulbecco’s Modified Eagle Medium F-12 (DMEM/F-12), Dulbecco’s phosphate-buffered saline (DPBS), penicillin/streptomycin, 0.25% Trypsin-EDTA (1×), and fetal bovine serum (FBS), 4′,6-diamidino-2-phenylindole (DAPI) were purchased from Thermo Fisher Scientific (Waltham, MA, USA). Sodium palmitate (PA), 3-(4,5-Dimethylthiazol-)2,5-diphenyl Tetrazolium Bromide (MTT), β-nicotinamide adenine dinucleotide (NADH), and tween-20 were purchased from Sigma Aldrich (Saint Louis, MO, USA). Sulfo-Nsuccinimidyl oleate (SSO) was purchased from Cayman Chemical (Ann Arbor, MI, USA). Dimethyl sulfoxide (DMSO), bovine serum albumin (BSA), and nonfat dried milk were purchased from PanReac AppliChem (Glenview, IL, USA). Before each experiment, all solutions were freshly prepared. Absolute ethanol, hydrochloric acid, and methanol were purchased from Carlo Erba Reagents (Cornaredo, Milan, Italy).

### 2.2. Cell Culture

H9c2 cardiomyoblast cells were obtained from the American Type Culture Collection (ATCC) (Manassas, VA, USA) (Cat# CRL-1446), cultured in Dulbecco’s Modified Eagle Medium/Nutrient Mixture F-12 (DMEM/F-12, Gibco, Thermo Fisher Scientific, Waltham, MA, USA) supplemented with 10% fetal bovine serum (FBS, Gibco), 1% penicillin/streptomycin (Thermo Fisher Scientific), and incubated in humidified atmosphere (5% CO_2_) at 37 °C. Upon reaching 80% cell confluence, H9c2 cardiomyocytes were digested at a 1:2 ratio using 0.25% Trypsin-EDTA (1×) (Gibco) according to the manufacturer’s instructions (ATCC). For experiments, cells were seeded in complete medium and incubated for 48 h at 37 °C, 5% CO_2_, as previously reported [16,18,19,20].

### 2.3. Cell Viability Assay

MTT assay was used to determine the effect of PSELT or I-PSELT on cell viability following PA exposure. H9c2 cells (5000 per well) were seeded in a 96-well plate and then treated with palmitate (PA) (from 100 to 500 µmol/L) for 24 h or vehicle (BSA) as control. PA was purchased from Sigma and dissolved to make a 10 mM stock solution with 10% fatty acid-free BSA [21]. Once the concentration for PA-induced cell death was established, H9c2 cardiomyocytes were exposed to PA (100 μmol/L) and PSELT (from 5 to 100 nmol/L) or its inactive form, indicated as inert-PSELT (I-PSELT), from 5 to 100 nmol/L, for 24 h. After treatments, 100 µL of 2 mg/mL MTT solution (Sigma Aldrich) was added to each well after removal of the culture medium, and then cells were incubated for 4 h at 37 °C, 5% CO_2_. Finally, MTT solution was replaced by DMSO, and formazan crystals were dissolved. The absorbance was measured at 570 nm using a microplate reader (Multiskan™ SkyHigh, Thermo Fisher Scientific Inc.). The means of the absorbance values of six wells in each experimental group were expressed as the percentage cell viability relative to the control cells. The experiment was repeated three independent times [16,18,19]. 

### 2.4. Lactate Dehydrogenase (LDH) Assessment

The entity of damage induced by PA in H9c2 cardiomyocytes was assessed by analyzing the levels of LDH released in the culture medium following the method of McQueen (1972) [22], and as previously indicated by Rocca et al. (2019) [23]. To analyze the levels of LDH released in the culture medium, H9c2 cells (100,000 per mL) were seeded in a 24-well plate and treated with vehicle (control), PA (100 μmol/L), PA + PSELT or PSELT (5 nmol/L) for 24 h. At the end of the treatments, 100 μL per well of the culture medium was used for LDH activity determination. The enzyme activity was evaluated spectrophotometrically using Multiskan™ SkyHigh (Thermo Fisher Scientific), following the method of McQueen (1972) [22]. The reaction velocity was determined by a decrease in absorbance at 340 nm resulting from the oxidation of NADH (indicative of LDH activity) that was expressed in IU/L [24].

### 2.5. Oil Red O Staining

Intracellular lipid accumulation was measured by Oil Red O staining. H9c2 cells were seeded in a 6-well plate, treated with vehicle, PA (100 μmol/L), PA + PSELT or PSELT (5 nmol/L) and incubated in a humidified atmosphere at 37 °C for 24 h. After the treatments, H9c2 cells were washed three times with DPBS and incubated with the Oil Red O kit according to the manufacturer’s instructions (#04–220923, Bio Optica, Milan, Italy). H9c2 cardiomyocytes were incubated with reagent A for 20 min, then washed and incubated with reagent B for 30 s followed by the addition of distilled water for 3 min. Nuclei were counterstained with hematoxylin provided by the kit [25,26,27]. After staining, cells were visualized under an Olympus BX41 microscope, and the images were taken with CSV1.14 software, using a CAM XC-30 for image acquisition.

### 2.6. Detection of Intracellular Reactive Oxygen Species (ROS) and Mitochondrial Superoxide Generation by MitoSOX

Intracellular reactive oxygen species (ROS) generation was measured using the cell-permeable probes CM-H_2_DCFDA (5-(and-6)-chloromethyl-2′,7′-dichlorodihydrofluorescein diacetate acetyl ester) (Thermo Fisher Scientific) and 2’,7’-dichlorodihydrofluorescein diacetate (H_2_DCFDA) (Thermo Fisher Scientific), while mitochondrial superoxide generation was evaluated by MitoSOX™ Red (Thermo Fisher #M36008, Waltham, MA, USA), a cell-permeable reagent that selectively targets mitochondria in live cells. 

For the detection of intracellular ROS, H9c2 cells (100,000 per well) were seeded on coverslips in 6-well plates and exposed to PA and PSELT, alone or in co-treatment for 24 h. At the end of the experimental protocol, H9c2 cardiomyocytes were incubated with 10 μmol/L CM-H_2_DCFDA at 37 °C for 30 min in the dark, and then the cells were carefully rinsed with DPBS and visualized under a fluorescence microscope (Olympus; 20× objective) [28,29]. For measuring the production of total ROS by flow cytometry, H9c2 cells were treated as above, collected, washed with DPBS, and stained with H2DCFDA. ROS were measured by flow cytometry (BD Fortessa X-20) according to the producer’s guidelines. The data were analyzed with FlowJo 10.8.1 software.

For mitochondrial superoxide generation, H9c2 cardiomyocytes were seeded on a coverslip in a 6-multiwell plate at a density of 100,000 cells per well and after 48 h, incubated with PA 100 (μmol/L) and PSELT (5 nmol/L) alone or in co-treatment for 24 h. At the end of the treatment, MitoSOX reagent was first dissolved in dimethylsulfoxide (5 mmol/L) and then diluted to 5 μmol/L in DMEM/F-12 phenol-free and serum-free medium [29]. A suitable amount of the solution was added to the cells followed by incubation for 10 min at 37 °C protected from light. Cells were then washed twice with DPBS. Fluorescence was detected using an Olympus fluorescence microscope (20× objective) and quantified using ImageJ 1.6 software (National Institutes of Health, Bethesda, MD, USA).

### 2.7. Immunofluorescence Analysis for CD36 Evaluation 

H9c2 cardiomyocytes were seeded on chamber slides (50,000 cells per chamber), incubated for 48 h at 37 °C, 5% CO_2_, and then treated with vehicle, PA (100 µmol/L), PA+ PSELT or PSELT (5 nmol/L) for 24 h. At the end of the treatment, H9c2 cells were washed three times with DPBS and fixed for 10 min with ice-cold methanol. Then, fixed cardiomyocytes were rinsed with cold-DPBS two times, and the permeabilization step was performed using 0.1% Triton X-100 in DPBS for 30 min at RT. Permeabilized H9c2 cells were then washed with DPBS following by blocking with 1% BSA in DPBS for 30 min at RT [16]. For immunofluorescence staining, H9c2 cells were incubated with a primary antibody against CD36 (diluted 1:200) for 2 h at room temperature and then stained with donkey anti-rabbit secondary antibody, Alexa Fluor 555 (diluted 1:1200), for 1 h at room temperature following the manufacturer’s instruction. The cells were then washed twice with DPBS and stained with DAPI for nuclei counterstaining. Images were obtained using an Olympus fluorescence BX41 microscope and acquired with CSV1.14 software, using a CAM XC-30 for acquisition. The fluorescence quantification was carried out using ImageJ 1.6 software. 

### 2.8. Short Interfering RNA (siRNA) Transfection for SELENOT Silencing

SELENOT gene silencing in H9c2 cardiomyocytes was performed as previously described by Rocca et al. (2022) [16]. Briefly, H9c2 cardiomyocytes (5000 per well) were seeded in 96-well plates and incubated for 48 h at 37 °C, 5% CO2. SELENOT siRNA (100 nmol/L) was transfected into H9c2 cardiomyocytes in serum-free medium using the Lipofectamine 2000 transfection reagent following the manufacturer’s instructions (Invitrogen, Thermo Fisher Scientific, Waltham, MA, USA). Negative control si-RNA (si-NC) was used to detect non-specific effects of siRNA delivery and to compare siRNA-treated samples. Both si-NC and siRNA for SELENOT were purchased from Santa Cruz Biotechnology. H9c2 cardiomyocytes were transfected in serum-free medium for 6 h, after which the medium was replaced with full-medium and cells were incubated for 36 h at 37 °C, 5% CO_2_. H9c2 cells were treated with PA (100 µmol/L) or co-treated with PA and increasing concentrations of PSELT (from 5 to 100 nmol/L) for 24 h. At the end of the treatments, the viability of the H9c2 cells was evaluated by MTT assay. The cell viability was reported as the percentage cell survival relative to the si-NC transfected cells in six wells for each experimental group [16]. The experiment was repeated three independent times.

### 2.9. Assessment of Mitochondrial Respiratory Function Using the Seahorse XF Analyzer

For the evaluation of mitochondrial respiration, real-time oxygen consumption rates (OCR) were determined using the Seahorse Extracellular Flux (XFe-96) (Seahorse Bioscience, Agilent Technologies, Inc), as previously described [30]. H9c2 cardiomyocytes were seeded at a density of 10,000 cells per well in a XF96 Seahorse microplate, incubated for 24 h in complete medium at 37 °C and 5% CO2, and then exposed to vehicle, PA (100 μmol/L), and PSELT (5 nmol/L), alone and in combination for 24 h. At the end of the treatments, media were replaced with pre-warmed Seahorse XF assay medium (Agilent Technologies, Inc.) supplemented with 10 mmol/L glucose and 1 mmol/L pyruvate and adjusted to pH 7.4. Cells were maintained in 175 μL of XF assay medium per well at 37 °C in a non-CO2 incubator for 1 h. During the incubation, 10 μmol/L oligomycin, 9 μmol/L FCCP, 10 μmol/L Rotenone, and 10 μmol/L antimycin A were loaded in XF assay medium into the injection ports in the XFe-96 sensor cartridge for OCR measurement. Data were analyzed by XFe-96 software, and measurements were normalized by the protein content, which was determined by Sulphorhodamine B assay, as previously described [30].

### 2.10. Western Blot

After the H9c2 cells were treated with vehicle, PA (100 μmol/L), PA + PSELT or PSELT (5 nmol/L) for 24 h, the cardiomyocytes were washed with DPBS and the total proteins were extracted using RIPA lysis buffer (Sigma Aldrich, St. Louis, MI, USA) supplemented with protease inhibitors [16]. Cell lysates were transferred in microcentrifuge tubes, incubated on ice for 30 min with intermittent mixing, and centrifuged at 12,000× *g* for 15 min at 4 °C. The supernatant was collected, and the protein concentration was determined by Bradford reagent using bovine serum albumin (BSA) as a standard. Equal amounts of protein (50 μg for all antigens and 30 μg for SELENOT) were loaded on 12% SDS-PAGE gel for N-terminus peroxisome proliferator-activated receptor-gamma coactivator-1 alpha (PGC-1α) and Superoxide dismutase 2 (SOD-2) and SELENOT; on 10% SDS-PAGE gel for Catalase (CAT); and on 8% SDS-PAGE gel for dynamin-related protein 1 (DRP-1) and optic atrophy 1 (OPA1). Gels were subjected to electrophoresis and transferred to polyvinyl difluoride membranes (GE Healthcare, Chicago, IL, USA). Membranes were blocked in 5% non-fat dried milk at room temperature for 1 h, washed three times with tris-buffered saline containing 0.1% Tween 20 (TBST), incubated overnight at 4 °C with primary specific antibodies for the antigens above mentioned, and diluted 1:1000 (for SELENOT and OPA-1), 1:500 (for PGC-1α, SOD-2, CAT) in TBST and 5% BSA, and 1:1000 for DRP-1 in TBST and non-fat dried milk 1%. β-actin antibody was used as a loading control. Following incubation with primary antibodies, the membranes were washed three times with TBST and then incubated with secondary antibodies peroxidase-conjugated at room temperature for 1 h (anti-mouse diluted 1:2000 and anti-rabbit diluted 1:3000) (Sigma Aldrich) in TBST containing 5% non-fat dried milk. Immunodetection was carried out using a chemiluminescence kit (Santa Cruz Biotechnology, Dallas, TX, USA) or Clarity Western ECL Substrate (Bio-rad, Hercules, CA, USA) when necessary. Densitometric analyses were performed using ImageJ 1.6 software (National Institutes of Health, Bethesda, MD, USA) as previously indicated [16,18,23].

### 2.11. Attenuated Total Reflectance Fourier-Transform Infrared (ATR-FTIR) Spectroscopic Measurements

For these analyses, H9c2 cells exposed to vehicle, PA (100 μmol/L), PA + PSELT or PSELT 5 nmol/L for 24 h were collected using trypsin, centrifuged (1500× *g*, 5 min), and then 1,000,000 cells were resuspended in 300 µL of the complete medium. The infrared spectra of live H9c2 cells were collected in the attenuated total reflectance (ATR) mode at 37 °C by using a Tensor II FTIR spectrometer (Bruker Optics, Ettlingen, Germany) equipped with a thermostated BioATR II sample holder and a mercury-cadmium-telluride detector. Then, 20 µL of the cell suspension was deposited on the ATR silicon crystal and left to equilibrate for 2 min before measurement. Spectra were recorded for 180 min (each 10 min) using the kinetic option in Opus acquisition software in order to maximize the absorption signal due to cell sedimentation on the crystal. Each spectrum was an average of 120 scans at 4 cm^−1^ spectral resolution. The background spectrum was recorded with the cell culture medium under the same experimental setup. At least three replicates for each cell type (grown independently) were measured. Spectral processing and analysis: the kinetic spectra of each measurement were baseline corrected with a rubber band function and averaged starting from the saturation stage using Opus 7 software. This average absorbance spectrum was further averaged with the replicas of the same cell type and normalized for the area under the amide II band (1597–1481 cm^−1^). The normalized mean spectra of each cell type were used in the calculation of the difference spectra. The statistical comparison between the normalized mean spectra of untreated and differently treated H9c2 cells was performed by using Student’s *t*-test (two-tailed, nonparametric Wilcoxon, 99% CI) at each wavenumber in Prism 5. The spectral regions where a significant difference in the absorption occurred (*p*-value < 0.0001) were considered in the discussion. The lipid/protein ratio was calculated from the area under the 3050–2800 cm^−1^ region (lipid content) and the 1700–1600 cm^−1^ region (protein content). The bandwidth of the CH2 symmetric band was measured at 75% of the height of the peak maximum from the baseline-corrected spectra.

### 2.12. Transmission Electron Microscopy (TEM) Analysis

H9c2 cardiomyocytes were seeded in 100 mm plates and upon reaching 80% confluence, they were treated with vehicle, PA (100 µmol/L), PA + PSELT (5 nmol/L) or PSELT (5 nmol/L). Cells were harvested using trypsin, centrifuged (1500× *g*, 5 min), and then fixed with 3% glutaraldehyde in 0.1 M phosphate buffer overnight at 4 °C. Post-fixation proceeded in buffered osmium tetroxide, followed by dehydration in a graded acetone series (30%, 50%, 70%, 90%, and 100%) and embedding in Epon812. Ultrathin sections (60–90 nm in thickness) were cut with a diamond knife, mounted on copper grids (G300 Cu), and imaged using a Jeol JEM 1400-Plus electron microscope operating at 80 kV. For each condition, at least 100 cells from randomly chosen fields were observed.

### 2.13. Statistical Analysis

Data, shown as means ± SEM, were analyzed by one-way ANOVA followed by Dunnett’s multiple comparison test and Newman–Keuls multiple comparison test (for post-ANOVA comparisons), and unpaired *t*-test when appropriate. Values with (*) *p* < 0.05, (**) *p* < 0.01, (***) *p* < 0.001, and (****) *p* < 0.0001 were considered statistically significant. The statistical analysis was conducted using Prism 5 (GraphPad Software, La Jolla, CA, USA).

## 3. Results

### 3.1. PSELT Mitigates PA-Induced Cytotoxicity and Lipid Accumulation in H9c2 Cardiomyocytes

**Figure 1:**
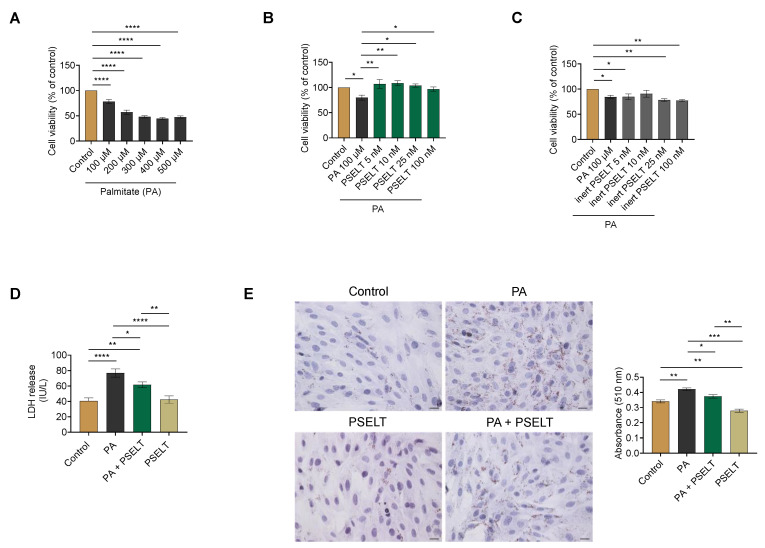
Effects of palmitate (PA) with or without PSELT on cell viability, cytotoxicity, and lipid accumulation in H9c2 cardiomyocytes. H9c2 cells were exposed to vehicle (Control) or increasing concentrations of (**A**) PA (100–500 µmol/L) or (**B**) PA 100 µmol/L + PSELT (5–100 nmol/L) or (**C**) PA 100 µmol/L + inert PSELT (I-PSELT) (5–100 nmol/L) for 24 h. The viability of H9c2 cells was determined using MTT assay and was expressed as the percentage of control cells. Results are reported as the mean ± SEM (*n* = 6 per group). Significant differences were detected by one-way ANOVA followed by Dunnett’s test, *p* < 0.05 (*); *p* < 0.01 (**); and *p* < 0.0001 (****) vs. the Control group. (**D**) Lactate dehydrogenase (LDH) release in the culture medium of H9c2 cardiomyocytes treated with vehicle, PA (100 µmol/L) and PSELT (5 nmol/L) alone or in co-treatment for 24 h. The LDH activity is expressed as IU/L. Data are shown as the mean ± SEM of six separate experiments. Significant differences were detected by one-way ANOVA and Newman-Keuls multiple comparison test, *p*< 0.05 (*); *p* < 0.01 (**); and *p* < 0.0001 (****). (**E**) Representative images of Oil Red O staining for lipid droplet assessment and relative quantification. H9c2 cardiomyocytes treated with vehicle (Control), PA 100 µmol/L, PA + PSELT 5 nmol/L or PSELT for 24 h. Scale bar: 25 µm. Quantification of the stained lipid droplets was performed by measuring the absorbance at 510 nm. Values are the mean ± SEM of three different experiments. *p* < 0.05 (*); *p* < 0.01 (**); *p* < 0.001 (***); and *p* < 0.0001 (****).

To determine the concentration range at which PA induces toxic action on cardiomyocytes in term of cell viability, H9c2 cells were treated with increasing concentrations of PA (100–500 μmol/L) for 24 h. MTT assay showed that, compared to control cells, PA dose-dependently decreased cardiomyocyte viability starting from 100 μmol/L (Figure 1A). After this first cardiotoxic dose of PA was established, H9c2 cells were exposed to PA (100 μmol/L) and co-treated with increasing concentrations of PSELT (5–100 nmol/L) for 24 h. The results indicated that PA induced a significant decrease in cell viability compared to the control cells, while PSELT was able to significantly mitigate PA-dependent cell death at each tested concentration, starting from 5 nmol/L (Figure 1B). Thus, the first effective concentration of PSELT (5 nmol/L) was considered for the subsequent analyses. Conversely, in the concentration range of 5–100 nmol/L, the inert counterpart of PSELT (inert-PSELT) was ineffective in counteracting PA-induced cell viability decrease (Figure 1C).

The effect of PSELT against PA-induced cytotoxicity was also evaluated by measuring, in the H9c2 culture medium, the enzymatic activity of LDH, whose release indicates damage of the cell membrane. As shown in Figure 1D, PA treatment significantly increased LDH release with respect to the control group, while H9c2 cells exposed to PA + PSELT exhibited a significant decrease in LDH activity compared to cells treated with PA alone. In the cells exposed to PSELT alone, no significant change in LDH levels was detected compared to control cells (Figure 1D). 

Oil Red O staining and relative spectrophotometric quantification were performed to measure the accumulation of intracellular lipids in H9c2 cells. This analysis revealed a significant increase in the intracellular lipid droplets in cardiomyocytes after PA exposure compared to control cells. However, in H9c2 cardiomyocytes treated with PA + PSELT, intracellular lipids were significantly reduced compared to PA alone (Figure 1E).

### 3.2. PSELT Protects H9c2 Cells against PA-Induced Oxidative Stress

**Figure 2:**
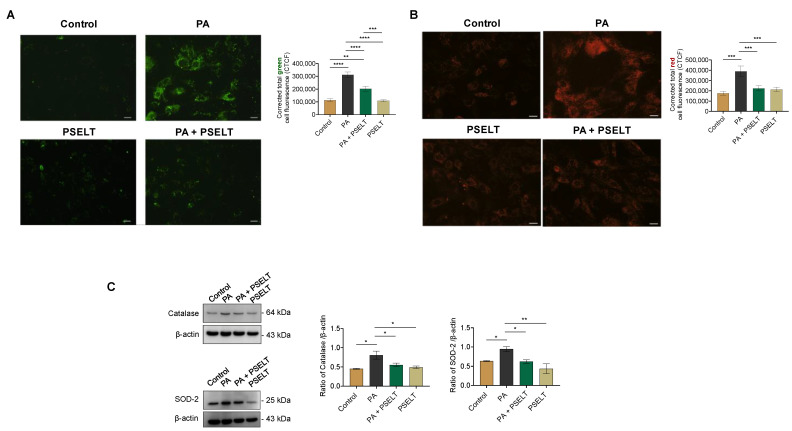
Effects of PSELT on PA-induced oxidative stress in H9c2 cardiomyocytes. (**A**) Representative images of a fluorescent probe (CM-H_2_ DCFDA) for ROS detection with relative fluorescence quantification. Data are expressed as the mean ± SEM (*n* = 3 different experiments). Scale bar: 25 µm. Significant differences in corrected total cell fluorescence (CTCF) were detected by one-way ANOVA and Newman-Keuls multiple comparison test *p* < 0.01 (**); *p* < 0.001 (***); and *p* < 0.0001 (****). (**B**) Superoxide (O_2_^−^) levels assessed by the MitoSOX fluorescent probe and quantification of the fluorescence intensity in H9c2 cardiomyocytes treated with vehicle (Control), PA 100 µmol/L, PA + PSELT 5 nmol/L or PSELT for 24 h. Scale bar: 25 µm. Data are expressed as the mean ± SEM (*n* = 3 different experiments). Significant differences in CTCF were detected by one-way ANOVA and Newman-Keuls multiple comparison test*, p <* 0.001 (***). Western blot analysis of (**C**) Catalase and SOD-2 in H9c2 cardiomyocytes treated with vehicle (Control), PA 100 µmol/L, PA + PSELT 5 nmol/L or PSELT for 24 h. Histograms represent the ratio of the densitometric analysis of protein:loading control. Statistical differences were analyzed by Newman-Keuls multiple comparison test (one-way ANOVA) *p* < 0.05 (*); *p* < 0.01 (**).

To evaluate whether PSELT could counteract PA-induced oxidative stress, we evaluated intracellular ROS generation by using the specific fluorescent probe CM-H_2_DCFDA, mitochondrial superoxide generation by MitoSOX-Red staining, and the expression levels of endogenous antioxidant enzymes. Figure 2A indicates that PA significantly increased ROS generation, as evidenced by the enhanced fluorescence intensity of the probe observed in PA-treated cells compared to control cells. PSELT significantly decreased this PA-induced fluorescence intensity, and PSELT alone did not generate any significant intracellular ROS compared to the control group. ROS determination by the H_2_DCFDA assay and measured by flow cytometry in H9c2 cells exposed to PA with or without PSELT reflected the same trend (Supplementary Figure S1).

Mitosox-Red staining was then conducted to detect mitochondrial superoxide generation in the H9c2 cardiomyocytes. As revealed by increased red fluorescence-stained cells in Figure 2B, PA treatment resulted in a significant increase in O_2_^−^ levels compared to those of control cells. PSELT treatment significantly reduced O_2_^−^ generation in the presence of PA compared to PA alone (Figure 2B). The cell oxidative status was also evaluated by assessing the expression levels of the endogenous antioxidant enzymes SOD-2 and CAT; Western blot and densitometric analyses of the H9c2 cell extracts showed that SOD-2 and CAT expression significantly increased in PA-treated cells with respect to the control group, whereas in the PA + PSELT group, the expression of both enzymes was significantly reduced compared to the group with PA alone (Figure 2C). 

### 3.3. PSELT Rescues the PA-Induced Reduction of Endogenous SELENOT Expression in H9c2 Cells, and Endogenous SELENOT Is Fundamental for PSELT-Induced Cell Protection against the PA Effect

**Figure 3:**
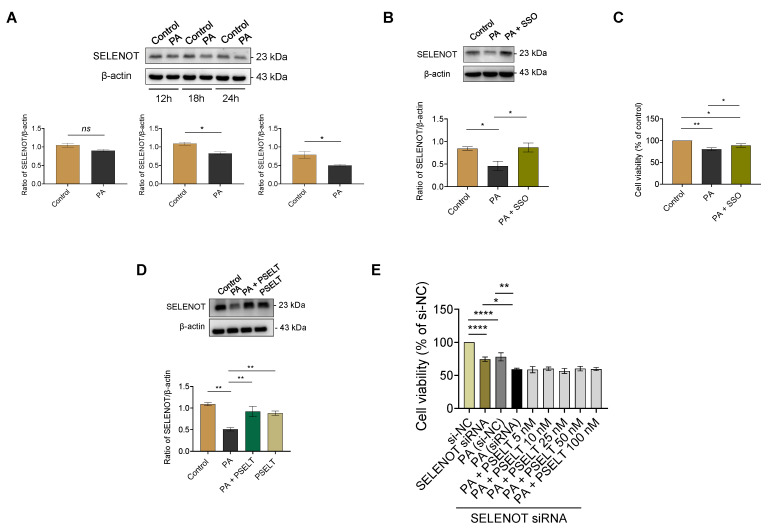
Effect of PA and PSELT on endogenous SELENOT expression and the role of SELENOT in PSELT-induced cell protection against PA in H9c2 cardiomyocytes. (**A**) Effect of PA on endogenous SELENOT expression at different times. Western blot analysis of SELENOT in H9c2 cardiomyocytes treated with vehicle (Control) or PA 100 µmol/L for 12 h, 18 h, and 24 h. Histograms represent the ratio of the densitometric analysis of protein:loading control. Data are expressed as the mean ± SEM, and significant differences were detected by *t*-tests; *p* < 0.05 (*). (**B**) Representative Western blot analysis of SELENOT in H9c2 cardiomyocytes treated with vehicle (Control), PA 100 µmol/L, or sulfosuccinimidyl oleate (SSO) 1 µmol/L for 1 h, an irreversible inhibitor of CD36 + PA, for an additional 24 h. Data are expressed as the mean ± SEM (*n* = 3 independent experiments). (**C**) MTT assay in H9c2 cardiomyocytes exposed to vehicle (Control), PA 100 µmol/L, or SSO 1 µmol/L for 1 h (an irreversible inhibitor of CD36) + PA for an additional 24 h. Cell viability was expressed as the percentage of control cells exposed only to vehicle (indicated as Control). (**D**) Western blot analysis of SELENOT in H9c2 cardiomyocytes treated with vehicle (Control), PA 100 µmol/L, PA + PSELT 5 nmol/L or PSELT for 24 h. Data are expressed as the mean ± SEM (*n* = 3 independent experiments). Histograms represent the ratio of the densitometric analysis of protein:loading control. (**E**) Effect of si-SELENOT gene silencing on cell viability in H9c2 cells treated with PA 100 µmol/L alone and in the presence of increasing concentrations of PSELT (5–100 nmol/L) for 24 h. Cell viability was evaluated using MTT assay and was expressed as the percentage of control cells transfected only with the si-RNA-negative control (indicated as si-NC). Results are presented as the mean ± SEM (*n* = 6 per group). Significant differences were detected by one-way ANOVA followed by Newman–Keuls multiple comparison test. *p* < 0.05 (*); *p* < 0.01 (**); and *p* < 0.0001 (****).

To evaluate whether PA could affect the expression of the endogenous SELENOT, we performed Western blot and relative densitometric analysis of SELENOT in H9c2 cells exposed to PA for 12, 18, and 24 h. As shown in Figure 3A, PA treatment reduced SELENOT levels in a time-dependent manner compared to the control, particularly at 18 and 24 h where the reduction of SELENOT expression was statistically significant. Then, we evaluated whether PA could decrease endogenous SELENOT expression through FAT/CD36 (cluster of differentiation 36/fatty acid translocase), the main transporter of fatty acids in the heart. To this aim, H9c2 cardiomyocytes were first treated for 1 h with 1 µmol/L of sulfo-N-succinimidyl oleate (SSO) [31]–an irreversible inhibitor of CD36 able to block CD36-mediated FA uptake–followed by exposure to PA for 24 h. As shown in Figure 3B, PA significantly reduced SELENOT expression compared to the control, while in the cells exposed to SSO + PA, SELENOT expression was preserved. To confirm that SSO was effective in preventing PA-induced cytotoxicity, we performed an MTT assay showing the ability of SSO to significantly mitigate PA-induced cell death in H9c2 cardiomyocytes compared to PA alone (Figure 3C). In order to evaluate the influence of exogenous PSELT on endogenous SELENOT expression during PA treatment, we carried out a Western blot analysis of H9c2 cells exposed to PA for 24 h in the presence or absence of PSELT. Figure 3D confirmed the ability of PA to reduce SELENOT expression compared to the control and indicated that PSELT significantly rescued the PA-induced reduction of SELENOT.

To establish the direct role of endogenous SELENOT in the cytoprotection mediated by exogenous PSELT during PA exposure, we tested the effect of SELENOT knockdown by using a SELENOT siRNA. We first observed that the transfection of the siRNA SELENOT (si-SELENOT) significantly reduced H9c2 cell viability compared to the control [i.e., cells transfected with control siRNA (si-NC)] (Figure 3E). As shown above, this analysis also showed that PA reduced cell viability in si-NC transfected cells compared to control cells (si-NC). Further, SELENOT knockdown worsened PA-induced cytotoxicity since the extent of the PA-induced cell death was higher in SELENOT-knockdown cells compared to the control cells exposed to PA [PA (si-NC) group] (Figure 3E). Additionally, none of the PSELT concentrations (5–100 nmol/L) in si-SELENOT-transfected cells exposed to PA were able to significantly mitigate PA-induced cell death compared with cells silenced for SELENOT and exposed only to PA (Figure 3E).

### 3.4. PSELT Reduces the PA-Dependent Upregulation of CD36 in H9c2 Cardiomyocytes 

**Figure 4:**
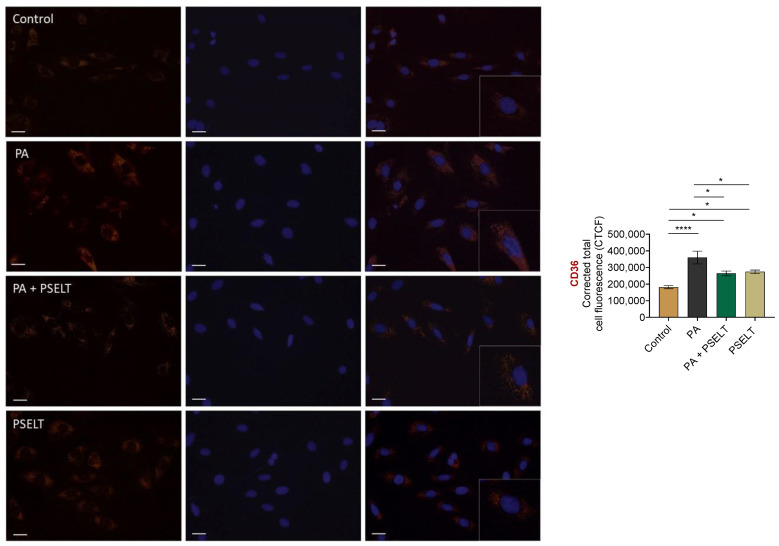
Effects of PSELT on CD36 expression in PA-treated H9c2 cardiomyocytes. Representative images of cardiomyocytes treated with vehicle or PA (100 µmol/L) and PSELT (5 nmol/L) alone or in co-treatment for 24 h. CD36 expression was assessed by using an anti-CD36 primary antibody (Thermo Fisher scientific) and a donkey anti-rabbit secondary antibody—Alexa Fluor™ 555 (red) (Invitrogen). Nuclei were counterstained with DAPI (blue). Scale bars: 25 μm. Data are expressed as the mean ± SEM (*n* = 3 different experiments). Significant differences in CTCF were detected by one-way ANOVA and Newman-Keuls multiple comparison test, *p* < 0.05 (*) and *p* < 0.0001 (****).

To evaluate whether PSELT could affect the expression levels of the fatty acid transporter CD36, we performed an immunofluorescence analysis on H9c2 cells exposed to PA and PSELT, alone and in co-treatment, for 24 h. As shown in Figure 4, PA significantly increased CD36 expression compared with control cells, as revealed by the enhanced fluorescence intensity in the PA group. Conversely, PSELT was able to significantly mitigate CD36 expression in H9c2 cardiomyocytes exposed to PA with respect to cells treated with PA alone, as revealed by a lower fluorescence intensity in the PA + PSELT group. Moreover, a slight but significant increase in fluorescence intensity was also detected in PSELT-treated H9c2 cells compared to control cells.

### 3.5. PSELT Mitigates the Detrimental Effects of PA on Mitochondrial Function, Biogenesis, and Dynamics

**Figure 5:**
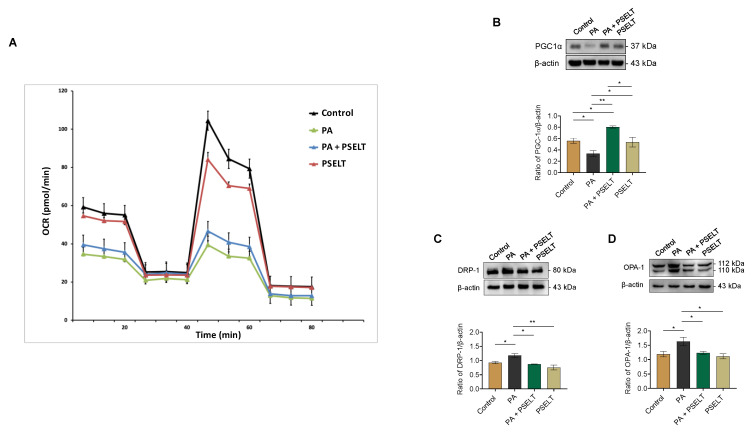
Effects of PSELT on mitochondrial respiration, biogenesis, and dynamics in H9c2 cells exposed to PA. (**A**) Evaluation of the mitochondrial respiratory capacity in H9c2 cardiomyocytes by Seahorse XF Analyzer after 24 h treatment with PA or PSELT alone or in combination and quantification of the oxygen consumption rates (OCR). Western blot analysis of (**B**) PGC-1α, (**C**) DRP-1, and (**D**) OPA-1 in H9c2 cardiomyocytes treated with vehicle (Control), PA 100 µmol/L, PA + PSELT 5 nmol/L or PSELT for 24 h. Histograms represent the ratio of the densitometric analysis of protein:loading control. Data are expressed as the mean ± SEM (*n* = 3 independent experiments). Significant differences were detected by one-way ANOVA and Newman-Keuls multiple comparison test. *p* < 0.05 (*); *p* < 0.01 (**).

To evaluate the effects of PSELT on mitochondrial function during PA treatment, we performed metabolic flux analysis using the Seahorse XFe96. In H9c2 cardiomyocytes, a dramatic reduction in the oxygen consumption rate (OCR) was observed after treatment with PA, whereas PSELT mitigated this effect (Figure 5A and Supplementary Figure S2).

We next investigated whether the protective action of PSELT could also be linked to mitochondrial biogenesis and dynamics; to this aim, we carried out Western blot analyses aimed at assessing specific markers involved in these molecular processes. Figure 5B shows that PA significantly reduced the levels of PGC1-α, a master regulator of mitochondrial biogenesis, compared to control cells. However, PSELT treatment significantly increased these levels in the presence of PA compared to cells exposed only to PA (Figure 5B). Moreover, we assessed whether PSELT could influence the expression levels of DRP-1, a key regulator of mitochondrial fission, and OPA-1, a gatekeeper of stress-sensitive mitochondrial fusion. Our results indicate that PA triggered a significant increase in both DRP-1 and OPA-1 expression compared to the control group, which was significantly reversed in the presence of PSELT (Figure 5C,D).

### 3.6. PSELT Mitigates PA-Dependent Ultrastructural Alterations in H9c2 Cardiomyocytes 

**Figure 6:**
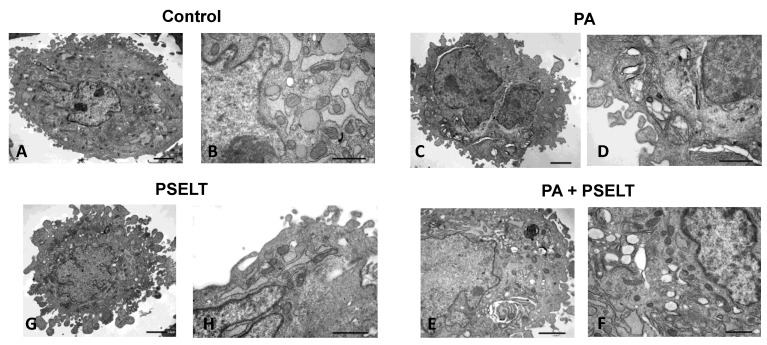
Representative images of transmission electron microscopy (TEM) regarding the effect of PA and PSELT, alone and in combination, on H9c2 cell ultrastructure. Transmission electron micrographs of control cells with intact architecture of cellular organelles, normal mitochondria with distinct cristea, and dilated ER profiles (**A**,**B**). PA treatment led to reorganization of the ER into concentrically whorled membranes that in some instances wrapped around degenerated mitochondria with swollen cristae (**C**,**D**). The beneficial effect of PSELT treatment was revealed by improved cell ultrastructure. ER membranes showing the ultrastructure of their naïve counterparts; mitochondria had highly organized cristae with regular thickness and electron density (**E**,**F**). PSELT treatment alone did not affect the regular arrangement of the intracellular structures and organelles (**G**,**H**).

To evaluate the protective action of PSELT against PA in H9c2 cardiomyocytes at the ultrastructural level, we carried out TEM analyses. Figure 6A,B shows that the cytoplasm of control cells was rich in ER, Golgi bodies, and mitochondria (see higher magnification images). Dilated cisternae of ER were found to be a typical component of control cells, while mitochondria possessed their typical cristae morphology and structure. After 24 h of PA treatment, the ER network reorganized into stacked and concentrically whorled membranes. The cristae disoriented in most of the mitochondria that also changed shape to oval or round, became swollen, and were often embedded in the ER (Figure 6C,D). Treatment with PSELT restored an apparent normal ultrastructure with no evidence of swelling or injury in cytoplasm and organelles. Most of the mitochondria showed regularly spaced lamellar cristae; the mitochondrial matrix also possessed the typical homogeneous staining pattern of modest electron density, see Figure 6E,F. PSELT treatment alone did not induce any significant effect on the structural and subcellular organization of the cells (Figure 6G,H).

### 3.7. PSELT Mitigates Specific FTIR Spectral Alterations Induced by PA in H9c2 Cardiomyocytes

**Figure 7:**
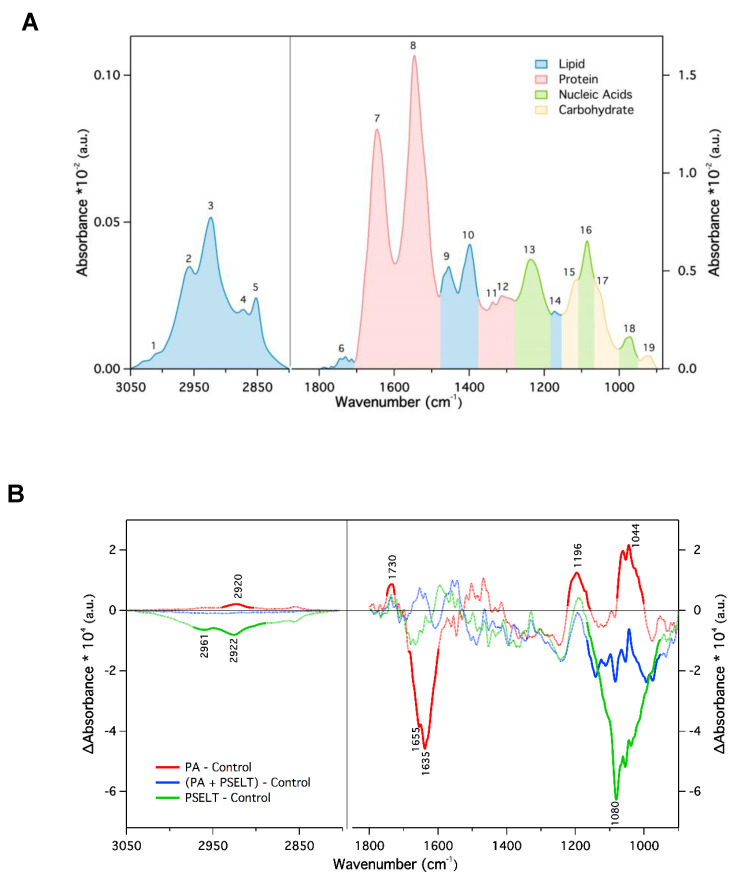
Effects of PSELT on FTIR spectral-related macromolecular changes of PA-treated H9c2 cardiomyocytes. (**A**) Representative mean ATR-FTIR spectrum of untreated H9c2 cells recorded at 37 °C in solution. The lipid region (3050–2800 cm^−1^) and the fingerprint region (1800–900 cm^−1^) are shown with the absorption of the different biomolecules. The labelled peaks indicate the absorbance of specific functional groups (**B**) Infrared difference spectra between differently treated cells H9c2 minus the mean spectrum obtained for untreated control H9c2 cells. Pre-processing spectral analysis included baseline correction and normalization to the area under the amide II band. The statistical significance (α = 0.01) was determined by using Student’s *t*-test at each wavenumber, and the spectral regions with *p*-values < 0.0001 are shown as thicker colored lines. (a.u. indicated arbitrary units).

Figure 7A shows a representative mean absorbance FTIR spectrum of H9c2 cells in aqueous solution in the spectral regions 3050–2800 and 1800–900 cm^−1^, which contain the signature of key biomolecules. The absorption in the first region originated mainly from the CH_2_ and CH_3_ stretching vibration (both symmetric and asymmetric) of the lipid acyl chain and reflects the molecular properties of cellular membranes. The second spectral region (fingerprint region) exhibited more complex spectral features where the contribution of the different biomolecules partially overlapped. The main peaks were due to lipids (1750–1715 cm^−1^ C = O stretching, and CH_2_ bending at 1470 cm^−1^), proteins (amide I, 1646 cm^−1^, amide II, 1546 cm^−1^, and amide III at 1300 cm^−1^), nucleic acids (asymmetric and symmetric phosphate stretching at 1125 and 1085 cm^−1^, respectively), and carbohydrate and glycogen (1155 and 1030 cm^−1^) [32,33]. For a more detailed description of the absorption peaks numbered in Figure 7A, see Table 1. Typically, variations of the IR bands may include peak shifts, linewidth changes, and variation in the peak intensity/area that can be correlated with specific modifications of the functional groups of the relevant molecules within the sample [34]. In particular, peak shifts and bandwidth provide structural and dynamical information whereas band intensity is related to the concentration of the corresponding functional groups of the biomolecules (according to the Beer–Lambert law).

Based on a comparison of the mean absorbance spectra of the four different cell samples analyzed, no variation of the band position was observed (Supplementary Figure S3), indicating that there were no significant structural changes. However, changes in the peak intensity were observed. 

To better visualize the effects of the different treatments on the molecular composition of H9c2 cardiomyocytes, we calculated the difference between the mean absorbance spectra of the treated cells minus the mean spectrum of the untreated cells representing our control sample (Figure 7B). The regions with the highest statistical significance (*p* < 0.0001) in absorbance were highlighted with a thicker line. Positive/negative peaks in the plot indicated higher/lower concentration of the molecular components within the cell. 

When we compared H9c2 cells treated with PA with control cells (red line), significant differences were observed in the amide I region, which reflects a decrease in protein concentration. The peaks positions at 1655 and 1635 cm^−1^ are associated with α-helix and β-sheet proteins [32]. Positive peaks were found around 1196 and 1046 cm^−1^. These regions are assigned due to the vibrations of specific groups of nucleic acids and carbohydrates and suggest changes in the content of such molecules. Positive peaks are also found for the lipid component at about 2920 and 1730 cm^−1^. These variations were also reflected in the lipid/protein ratio for the two samples, which increased from 0.200 ± 0.079 (control) to 0.337 ± 0.043 for the PA sample. Moreover, the bandwidth of the CH_2_ symmetric band at 2852 cm^−1^ decreased from 16.747 ± 0.003 cm^−1^ (control) to 16.030 ± 0.401 cm^−1^ (PA). Such a reduction suggests a decrease in cell membrane fluidity under PA treatment. When the cells were co-treated with PSELT (blue line), the difference in absorption almost vanished below 1200 cm^−1^ and only a reduction in the components related to nucleic acids and carbohydrates was observed. 

Finally, the effect of PSELT alone on the cells (green line) involved a reduction of the lipid content (see the negative peak at 2922 cm^−1^) and also of the bands related to nucleic acids and carbohydrates. Regarding the effects induced by PA in the region between 1165 and 955 cm^−1^, PSELT co-treatment mitigated the PA-dependent increase (blue line).

## 4. Discussion

Cardiac lipotoxicity can induce cell dysfunction and cell death, thus increasing both atherosclerotic coronary heart disease and HF, which represent important contributors to cardiovascular complications among obese individuals [6]. Although diverse studies have been conducted in this field, it is mandatory to improve our knowledge on the molecular mechanisms that drive cardiac lipotoxicity in order to characterize specific approaches to minimize obesity-related cardiac complications. In the present study, we employed H9c2 cardiomyocytes exposed to PA, a widely used in vitro model for recapitulating the cardiac harmful effects of consuming a high-fat diet, and provided novel evidence on the beneficial action of the antioxidant SELENOT mimetic peptide (PSELT) against cardiomyocyte damage induced by lipotoxicity.

### 4.1. PSELT Exerts Protective Effects against PA-Induced Cytotoxicity and Lipotoxicity through Its Redox Site Containing the Sec Residue

Several reports indicate that PA causes lipotoxicity and cell dysfunction in many cell types, including cardiomyocytes [36,37,38,39,40]. Therefore, we exposed H9c2 cells, which are widely used as a cell line to model cardiomyocytes in vitro due to their biochemical, morphological, and electrical/hormonal properties [41,42], to PA in order to model hyperlipidaemia in vitro and to focus on the mechanism affecting the myocardium (i.e., lipotoxicity), which is implicated in the pathogenesis of HF in obesity. In line with previous studies, our results showed first that PA caused cardiomyocyte death in a dose-dependent manner, indicating that the in vitro model of cytotoxicity was successfully established. In accordance, the use of PA represents the most commonly method to induce cardiac lipotoxicity in in vitro systems [43,44].

Our previous in vivo and in vitro data demonstrated that SELENOT exerts a crucial role in preserving redox and ER homeostasis and is essential for cardiomyocyte differentiation and protection, as well as for glucose metabolism through its ability to regulate insulin production/secretion [12,13,14,15,16]. Therefore, we hypothesized that SELENOT could play a role in protecting cardiomyocytes exposed to a dysmetabolic condition generated by lipid overload. To address this issue, we took advantage of a selective SELENOT-derived small peptide (PSELT) able to mimic the activity of the full-length protein through its CVSU redox motif, as previously reported by our groups in different pathophysiological contexts [15,16,17]. In this regard, therapeutic peptides are emerging as very promising tools due to their ability to selectively target specific molecules and pathways, which may circumvent some limitations of the conventional therapeutics such as those related to the use of a full-length protein in its recombinant form [45].

Our results indicated that PSELT counteracts lipotoxic cardiomyocyte death through its redox active site, as an analogous control peptide lacking the Sec residue in the catalytic site (i.e., inert PSELT) was ineffective, indicating that the cytoprotective action of PSELT is attributable to the Sec residue in the CVSU motif. The protective effects of PSELT against PA-induced lipotoxicity were also confirmed by its ability to reduce the release of LDH in the culture medium (i.e., an important indicator of cytotoxicity and membrane damage) and to counteract intracellular lipid droplets [46]. 

### 4.2. PSELT Counteracts PA-Induced Oxidative Stress and the Reduction of Endogenous SELENOT 

It is widely known that oxidative stress plays a key role in the onset and progression of several multifactorial diseases, including obesity-related cardiovascular disorders [47,48,49]. Therefore, selective pharmacological interventions aimed at inhibiting ROS overproduction could represent suitable strategies to mitigate lipotoxic cardiomyocyte death and cardiac dysfunction [50]. Here, we report that PSELT mitigated intracellular ROS production and the mitochondrial superoxide generation provoked by PA and decreased the PA-dependent activation of key endogenous antioxidant enzymes, such as SOD and catalase. It has been reported that an increase in ROS production induces lipid peroxidation, stimulating the activation of antioxidant defenses and inducing redox homeostasis imbalance [51,52]. For instance, the decrease in glutathione (GSH) reductase and glutathione peroxidase (GPX) activities in the presence of PA may activate the antioxidant enzymes SOD and CAT as the result of an adaptive response employed by the cell to counteract the lipotoxic stress condition [53]. The increase in SOD-2 levels found in our study during PA treatment could be due to the augmented production of mitochondrial superoxide, which in turn may contribute to ROS generation. The increase in CAT during PA could be linked to excessive hydrogen peroxide production due to the increase in SOD-2 levels. Therefore, our results suggest that PSELT may induce direct antioxidant activity and a consequent equilibrium of the redox status by rebalancing the levels of ROS-metabolizing enzymes. It is known that a close relationship between increased levels of cytosolic/mitochondrial Ca^2+^ and excessive ROS generation promoting cardiomyocyte and endothelial dysfunction exists [54,55]. It is also widely accepted that PA triggers oxidative stress, leading to disrupted redox-dependent regulatory mechanisms of ER homeostasis and ER stress resulting in Ca^2+^ dysregulation, which may actively participate in apoptotic cell death [56]. Although we did not investigate the cytosolic and mitochondrial Ca^2+^ overload and its potential role in PA-cardiac lipotoxicity, literature evidence has reported Ca^2+^ overload in cardiomyoctes during PA exposure, even if at higher doses compared to those used in our study [21,57]. Considering that SELENOT crucially modulates ER thiol redox balance and contributes to Ca^2+^ signaling by modulating Ca^2+^ flux into and from the ER lumen and by a redox mechanism involving thiol groups in calcium channels and pumps [12,58], it is possible that PSELT and/or SELENOT can be functionally involved in Ca^2+^ regulation for mediating their beneficial action during lipid overload conditions.

The ability of PSELT to mitigate the alteration of cardiomyocyte redox status following PA prompted us to investigate whether PA could also affect the expression of SELENOT (i.e., another key endogenous antioxidant enzyme) and to evaluate the functional role of endogenous SELENOT in the exogenous PSELT-mediated cytoprotection. Our results showed first that PA time-dependently decreased SELENOT expression, indicating that the lipid overload-induced cardiomyocyte injury may depend, at least in part, on the decrease in a selenoprotein faithfully involved in ER homeostasis and exerting a crucial protective role in cardiomyocytes, which also acts as a redox-sensing protein [12,15,16]. We then determined whether PA-dependent SELENOT downregulation could be mediated by FAT/CD36, which plays a pivotal role in the uptake of long-chain fatty acids under both physiological and pathological conditions and is responsible for more than 70% of fatty acid uptake/oxidation in the heart [59,60]. To this aim, we employed SSO, an irreversible inhibitor of FAT/CD36 blocking the uptake of fatty acids, and found that pre-treatment with this compound not only prevented SELENOT downregulation in cells exposed to PA but also inhibited lipotoxic cell death, indicating that PA can affect endogenous SELENOT expression through CD36, indicating this selenoprotein as a novel molecular actor in CD36 signaling for which a crucial role in lipid-overloaded hearts has been reported in several studies [[61] and references therein]. Intriguingly, PSELT rescued SELENOT protein expression after PA treatment, indicating important physiological crosstalk between the exogenous peptide and the endogenous protein. To further corroborate this hypothesis, we assessed the effect of PSELT during PA treatment in SELENOT-silenced cardiomyocytes. The results showed that H9c2 cell viability was compromised by SELENOT deficiency per se, a phenomenon that was worsened in SELENOT-silenced cells exposed to PA, confirming the essential role of this selenoprotein in cardiomyocyte survival and function [15,16]. However, PSELT addition did not mitigate lipotoxic damage in SELENOT-silenced cardiomyocytes, suggesting that SELENOT is required for PSELT action and that the combined action of the protein and the peptide is fundamental to protect cardiomyocytes from the lipotoxic insult. The ability of exogenous PSELT to prevent PA-induced SELENOT down-regulation could be of particular interest, as SELENOT not only plays a crucial role in cardiomyocyte differentiation and protection but also represents an essential protein for life, as revealed by the fact that it is the only ER selenoprotein whose gene disruption leads to early embryonic lethality, it is the most highly conserved selenoprotein throughout evolution, and it represents one of the highest-priority selenoproteins [62,63]. 

Based on the ability of CD36 to affect SELENOT expression and to potentially use this mechanism, among others, for mediating cardiac lipotoxicity, we wondered whether PSELT could influence CD36 expression during PA exposure. Our immunofluorescence analysis revealed that the peptide mitigated the PA-induced upregulation of CD36, indicating that PSELT can protect cardiomyocytes and prevent SELENOT decrease during PA treatment by also reducing the uptake of PA through this transporter. Although we currently do not have the possibility to unequivocally demonstrate that PSELT may influence the biological activity of CD36 through a direct binding to this transporter since a specific antibody against the portion of SELENOT encompassing the PSELT sequence does not exist, our immunofluorescence analysis, which also indicated that PSELT was able to stimulate CD36 under basal conditions (without PA), suggested that the regulation of CD36 expression through direct binding may exist. On the other hand, we cannot exclude that PSELT, acting intracellularly [16,17], may regulate SELENOT expression during stressful conditions induced by PA, making the PSELT/SELENOT crosstalk particularly complicated.

### 4.3. PSELT Improves Mitochondrial Ultrastructure and Function in Terms of Respiration, Biogenesis, and Dynamics in PA-Treated Cardiomyocytes

There is mounting evidence that maintaining mitochondrial integrity and function is crucial for cardiac cells [64,65]. Here, we determined the effect of PA with or without PSELT on mitochondrial respiration in H9c2 cardiomyocytes. Our data showing a decrease in the oxygen consumption rate in PA-treated cells are consistent with the ability of PA to act as a partial inhibitor of the electron transport chain and to induce oxidative imbalance in mitochondria [21,66]. Interestingly, PSELT improved mitochondrial respiration and mitigated PA-induced mitochondrial respiratory dysfunction. 

Mitochondrial alterations can be also related to their biogenesis and dynamic deficiency, which plays a critical role in lipotoxicity and lipid overload-induced metabolic disorders [67]. To further delineate which of these mechanisms are involved in PSELT-dependent mitochondrial protection during PA deleterious effects, we first analyzed the expression levels of PGC1-α, a transcriptional coactivator that plays a key regulatory role in mitochondrial biogenesis. Consistent with several studies [68,69], our results showed that PA markedly reduced PGC1-α expression, an effect that was completely reversed by PSELT, suggesting a potential action of the peptide in promoting mitochondrial biogenesis. Consolidated data have also indicated the presence of cross-regulatory circuits coordinating mitophagy, mitochondrial dynamics, and mitochondrial biogenesis aimed at maintaining the quantity and quality of the mitochondrial network [70]. Therefore, we also studied the potential action of PSELT in mitochondrial dynamics by assessing the expression levels of DRP1, an essential regulator of mitochondrial fission and OPA-1, a critical mediator of the fusion process. In our study, PA induced an increase in both markers, indicating a significant imbalance of fission/fusion that will inevitably lead to mitochondrial dysfunction. The increased mitochondrial fusion observed during PA exposure could represent an attempt of the cell to address and mitigate lipotoxic stimulus, while the increased fission could be required for generating new mitochondria on the one hand, and for removing injured mitochondria through apoptosis on the other hand, to control mitochondrial quality [71]. Although different stimuli, including the excess of lipids, can differentially regulate mitochondrial dynamics, activating or inhibiting mitochondrial fission/fusion depending on the context [72], our data showed that PSELT may restore the levels of both DRP-1 and OPA-1 to normal conditions, thus counteracting PA-induced mitochondrial alterations by re-establishing mitochondrial fission/fusion balance. 

Important evidence also highlighted the active participation of ER in mitochondrial division establishing a new model linking mitochondrial dynamics and cell death [71,73]. Moreover, saturated fatty acids have been reported to be lipotoxic in cardiomyocytes due to their generation of ER stress [3,74]. Thus, in this study we evaluated ultrastructural changes during PA and PSELT treatments, focusing on mitochondrial morphology and the ER network. Our TEM analyses showed impaired mitochondrial morphology characterized by the swelling of mitochondria and injured mitochondrial cristae as well as mitochondrial embedding within the ER and the presence of alterations in the ER network in cardiomyocytes exposed to PA. These findings are in line with several studies reporting ultrastructural changes secondary to PA exposure that are mainly linked to ROS overproduction and oxidative stress [47,75,76,77]. Interestingly, PSELT not only improved PA-induced cristae remodeling but also restored the conventional mitochondrial ultrastructure and promoted the ER network, reinforcing the hypothesis that the peptide can protect cardiomyocytes against PA by ameliorating the mitochondrial structure and function and ER network. Notably, we previously demonstrated that SELENOT localizes in the cardiac ER and plays a crucial role in the regulation of ER proteostasis, acting as a “guardian” of ER homeostasis through its redox center [12,15]. Therefore, the ability of PSELT to cross the plasma membrane and potentially localize/target ER should be regarded with particular interest in the context of key functional cooperation between exogenous PSELT and endogenous SELENOT in ER homeostasis under both physiological and lipid overload conditions.

### 4.4. PSELT Attenuates FTIR Spectral-Related Macromolecular Changes Induced by PA in H9c2 Cardiomyocytes

The beneficial action of PSELT against PA prompted us to deepen our understanding of the effect of the peptide on the macromolecular content by a spectroscopic characterization. FTIR spectroscopy analysis revealed positive peaks in the lipid component of PA-treated cells, confirming the ability of saturated fatty acids to induce lipid overload and cardiac lipotoxicity [78,79,80]. We also found that PA negatively affected the content of the α-helix and β-sheet structures of proteins; this effect is likely related to the oxidative burst and ER stress generated by PA that may activate UPR, impairing protein synthesis. This hypothesis is consistent with recent findings according to which PA causes a decline in protein synthesis in skeletal muscle by inducting ER stress [46]. We then found that PA also promoted a substantial increase in the content of nucleic acids, which could reflect a first-line response adopted by cardiomyocytes against cellular damage, aimed at reconstructing new mitochondria and microsomes. This hypothesis is in line with previous studies indicating the capability of myocardial tissue to enhance the RNA/DNA concentration for the early renewal of injured cell material following specific insults, such as myocardial infarction [81]. 

Intriguingly, PSELT markedly reduced PA-induced lipid accumulation, further confirming the direct action of the peptide in reducing the accumulation of lipid droplets, and mitigated the PA-dependent reduction in protein synthesis and increase in nucleic acids. These findings agree with our recent studies showing that SELENOT, as a novel subunit of the oligosaccharyl transferase (OST) complex, is crucial for the regulation of ER proteostasis, hormone *N*-glycosylation, folding, and secretion [11,13]. Additionally, TEM analyses showing the ability of PSELT to improve the ER ultrastructure and network, together with our recent work indicating that PSELT exerts cardioprotection by relieving ER stress in a rat model of MI/R [16], corroborate the idea that this peptide may preserve the ER structure and function in cardiomyocytes and thus counteract PA-dependent protein synthesis decline.

Another aspect of our spectroscopic findings refers to a possible decrease in membrane fluidity observed in H9c2 cells treated with PA, which could be related to a decrease in the content of cholesterol, unsaturated and saturated lipids, and proteins, as well as to the action of free radicals that can promote molecular oxidation through different mechanisms [82]. Interestingly, PSELT mitigated the loss in cardiomyocyte membrane fluidity, corroborating its ability to restore the lipid profile and to exert antioxidant defense. Indeed, consolidated evidence reported the ability of oxygen free radicals to alter the cell membrane fluidity. Particularly, different oxyradical generating systems can affect the function of cardiac membranes by decreasing the phospholipid N-methylation activity, which is known to determine the membrane fluidity [83]. This effect may represent the result of lipid peroxidation that occurs when a free radical oxidizes an unsaturated lipid chain, leading to the formation of a hydroperoxidized lipid and an alkyl radical, thus altering membrane integrity, fluidity, and function [84,85]. Together, these data further delineate the biological significance of SELENOT in controlling the homoeostasis of cardiomyocytes during oxidative stress triggered by various insults including lipotoxicity.

## 5. Conclusions

**Figure 8:**
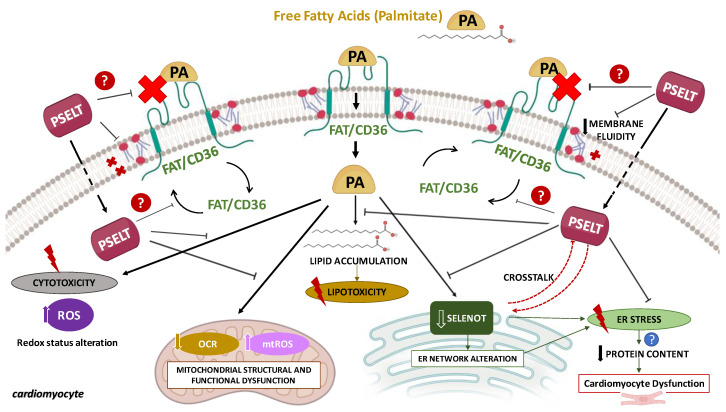
Schematic molecular mechanisms underlying the protective effect of the redox-active motif of SELENOT (PSELT) against palmitate-induced lipotoxicity in cardiomyocytes. ER: endoplasmic reticulum; FAT/CD36: cluster of differentiation 36/fatty acid translocase; OCR: oxygen consumption rate; PA: palmitate; mtROS: mitochondrial reactive oxygen species; ROS: reactive oxygen species; SELENOT: selenoprotein T. For further explanation, see text.

In conclusion, this study reports the ability of the SELENOT mimetic PSELT to inhibit PA-provoked detrimental effects in H9c2 cardiomyocytes by counteracting cell death, lipid accumulation, redox alteration, CD36 upregulation, and mitochondrial and ER dysfunction (Figure 8). Our results also indicate that PSELT can preserve PA-dependent alterations in the cellular macromolecular content and membrane fluidity. Interestingly, these findings suggest that exogenous PSELT requires endogenous SELENOT–a crucial protein for cardiomyocyte differentiation and protection–to exert its beneficial action during lipotoxicity, further delineating the biological significance of SELENOT in cardiomyocytes and highlighting important physiological crosstalk between the exogenous peptide and the endogenous protein (Figure 8).

## Figures and Tables

**Table 1:** Band Assignments of the Main Peak Absorption of Cells in the 3050–900 cm^−1^ IR Spectral Region [34,35].

Peak Number	Wavenumber (cm^−1^)	Assignment of Functional Groups
1	3010	Olefinic = CH stretching vibration (unsaturated lipids)
2	2957	CH_3_ asymmetric stretching (lipids)
3	2923	CH_2_ asymmetric stretching (lipids)
4	2872	CH_3_ symmetric stretching (proteins and lipids)
5	2852	CH_2_ symmetric stretching (mainly lipids)
6	1750–1715	C=O stretching (ester functional groups in lipids)
7	1646	Amide I (protein C=O stretching)
8	1546	Amide II (protein NH bending, CN stretching)
9	1456	CH_2_ bending (mainly lipids)
10	1399	COO^–^ symmetric stretching (fatty acids)
11	1337	CH_3_ symmetric bending (lipids)
12	1313	CH_2_ wagging (lipids)
13	1236	PO_2_^–^ asymmetric stretching, fully hydrogen-bonded (mainly nucleic acids)
14	1173	CO–O–C asymmetric stretching (ester bonds in cholesteryl esters)
15	1116	Ribose ring vibrations (RNA)
16	1086	PO_2_^–^ symmetric stretching (nucleic acids and phospholipids)
17	1050	C–O stretching (polysaccharides, glycogen)
18	971	C–N^±^–C stretching (nucleic acids)
19	924	Ribose ring vibrations (RNA)

## Data Availability

The data presented in this study are available in the article.

## Supplementary Materials

The following supporting information can be downloaded at https://www.mdpi.com/article/10.3390/cells12071042/s1. Figure S1: PSELT mitigates PA-induced intracellular ROS generation. Intracellular ROS levels assessed by H_2_DCFDA molecular probe in H9c2 cells treated with vehicle (Control), palmitate (PA), PA and PSELT (PA + PSELT), or PSELT only (PSELT). (A) Representative DCFH fluorescence intensity evaluated by flow cytometry (B) DCFH fluorescent intensity analysis. Values are the mean ± SD of three different experiments. *p* < 0.01 (**) and *p* < 0.0001 (****); Figure S2: PSELT mitigates the detrimental effect of palmitate (PA) on mitochondrial respiration. A significant reduction in basal respiration was observed in cardiomyocytes stimulated with PA, whereas this effect was mitigated by PSELT. Data shown are the mean ± SEM of 2 independent experiments performed in triplicate. * *p* < 0.05; *** *p* < 0.001 indicates significance, all relative to the control cells; Figure S3: ATR-FTIR mean spectra of control H9c2 cells (Control) and differently treated cells, specifically with palmitate (PA), sodium palmitate and peptide (PA + PSELT), or peptide only (PSELT). The cells were dispersed in their culture medium at a concentration of 10^−6^/300 µL and maintained at 37 °C in the BioATR II sample holder. The spectral resolution is 4 cm^−1^. The spectra were normalized to the area under the amide II band (1597–1481 cm^−1^).

## Abbreviations

CAT	catalase
CM-H_2_DCFDA	(5-(and-6)-chloromethyl-2′,7′-dichlorodihydrofluorescein diacetate acetyl ester)
CTCF	corrected total cell fluorescence
CVD	cardiovascular diseases
DRP-1	dynamin-related protein 1
ER	endoplasmic reticulum
FAs	fatty acids
FAT/CD36	cluster of differentiation 36/fatty acid translocase
ATR-FTIR	Attenuated total Reflectance-Fourier-transform infrared spectroscopy
GSH	glutathione
GPX	glutathione peroxidase
HF	heart failure
I-PSELT	inert selenoprotein T-derived peptide 43–52
LDH	lactate dehydrogenase
MI/R	myocardial ischemia/reperfusion
OCR	oxygen consumption rate
OPA-1	optic Atrophy 1
OST	oligosaccharyl transferase
PA	palmitate
PGC1-α	peroxisome proliferator-activated receptor-gamma coactivator1-α
PSELT	selenoprotein T-derived peptide 43–52
ROS	reactive oxygen species
Sec, U	selenocysteine
SELENOT	selenoprotein T
siRNA	short interfering RNA
SOD	superoxide dismutase
SSO	sulfo-N-succinimidyl oleate
UPR	unfolded protein response
