# Supplementary figures and images for: Palmitate-Induced Cardiac Lipotoxicity Is Relieved by the Redox-Active Motif of SELENOT through Improving Mitochondrial Function and Regulating Metabolic State
Source: Cells. 2023 Mar 29;12(7):1042. doi: 10.3390/cells12071042 (PMC10093731; doi:10.3390/cells12071042)

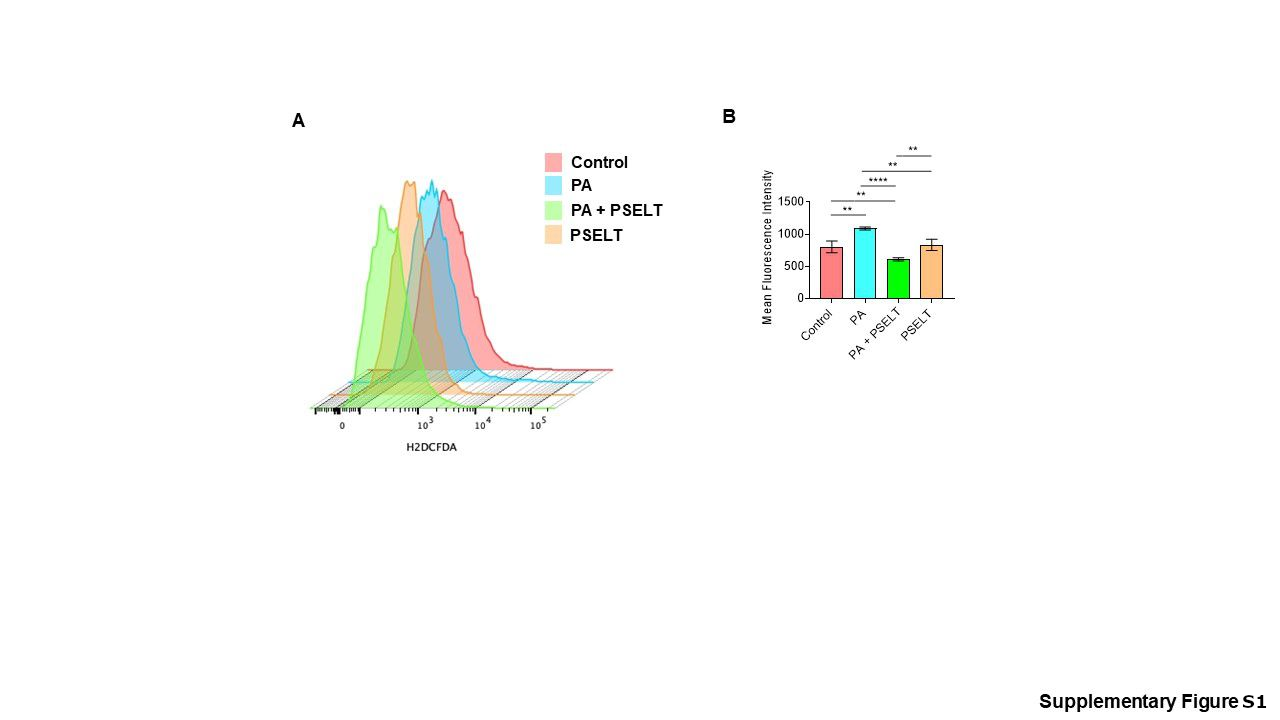

Supplement: Supplementary file 1 [file cells-12-01042-s001.zip › Supplementary Figure S1.tiff]

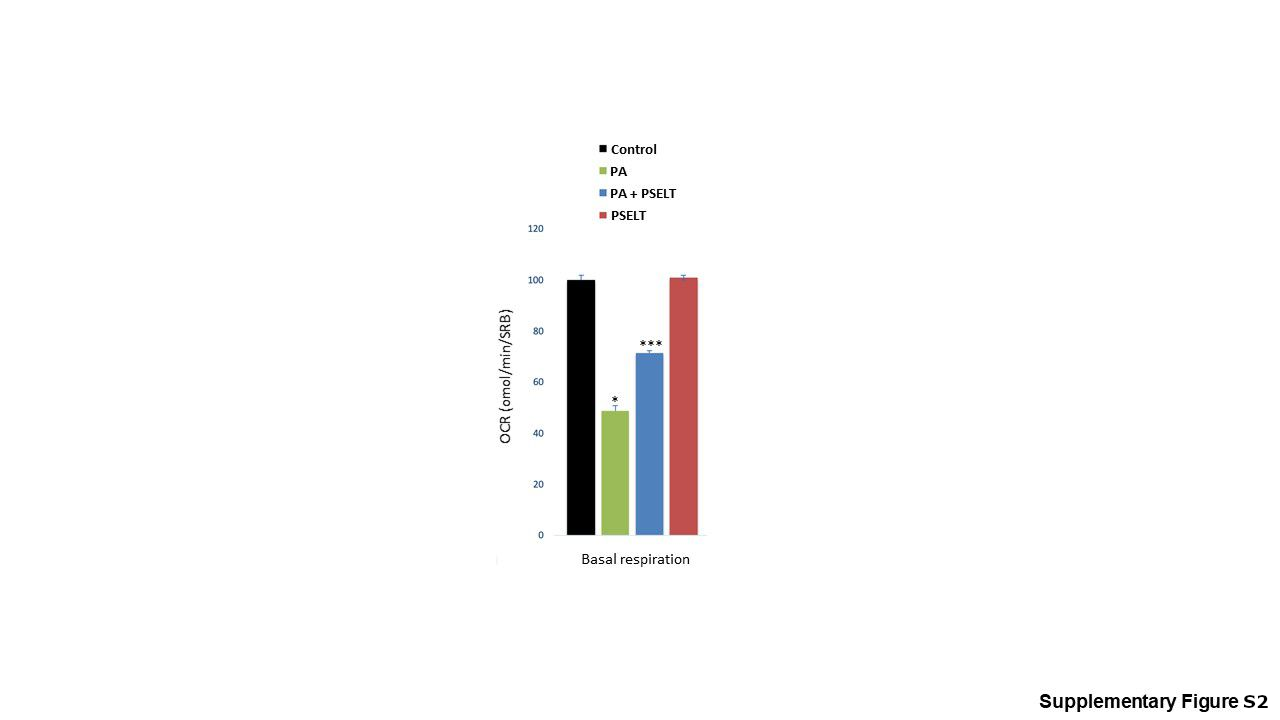

Supplement: Supplementary file 1 [file cells-12-01042-s001.zip › Supplementary Figure S2.tiff]

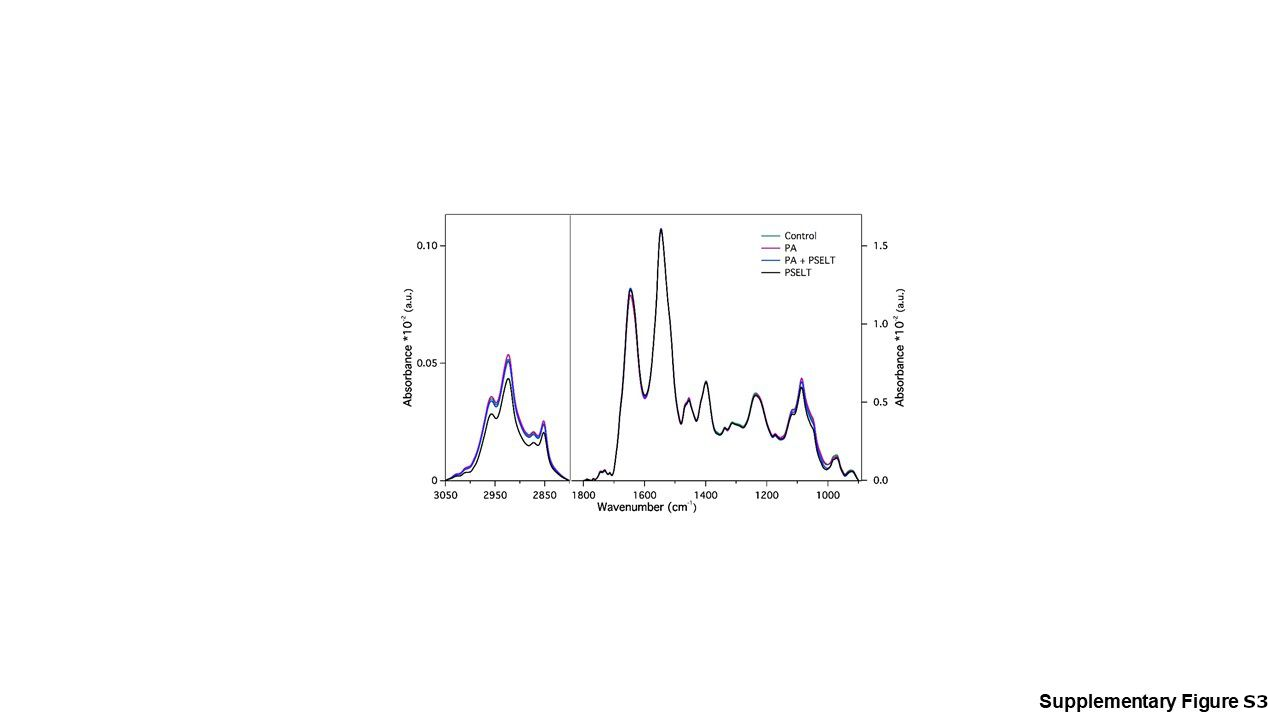

Supplement: Supplementary file 1 [file cells-12-01042-s001.zip › Supplementary Figure S3.tiff]
